# Sporotrichosis, Plain of Jars, Lao People's Democratic Republic

**DOI:** 10.3201/eid1109.050240

**Published:** 2005-09

**Authors:** Paul N. Newton, Wen-Hung Chung, Rattanaphone Phetsouvanh, Nicholas J. White

**Affiliations:** * Mahosot Hospital, Vientiane, Lao People's Democratic Republic;; † Churchill Hospital, Oxford, United Kingdom;; ‡ Chang Gung Memorial Hospital, Taipei, Taiwan, Republic of China;; § Mahidol University, Bangkok, Thailand

**Keywords:** sporotrichosis, *Sporothrix schenckii*, Lao PDR, Plain of Jars, itraconazole, rice miller, letter

**To the Editor:** In May 2003, a previously healthy, 42-year-old rice farmer and miller, living on the Plain of Jars (Xieng Khuang Province) in northeast Lao People's Democratic Republic (PDR) (Laos), dehusked and polished glutinous rice in her hand-operated rice mill. While milling, her hand slipped, removing the skin covering the interpharyngeal joint of her right index finger, on a dusty, wooden part of the machine. She did not recall the implantation of a wood splinter. During the following 4 weeks, multiple firm, erythematous lesions developed, which were not tender, fluctuant, or itchy, at the site of the injury and on the medial and anterior aspects of the lower and upper arm (Figure). The lesions spread proximally from the site of injury, but they remained confined to her right arm. She had no fever, and no lymphadenopathy developed. Her household had no domestic animals, including cats. No systemic disease developed, and she showed no evidence of immunosuppression, diabetes, or alcoholism. While waiting for a diagnosis, she persuaded a surgeon to excise all the lesions, but they soon recurred. She believed that the only solution would be to have her arm amputated. Initial biopsy specimens demonstrated no organisms and showed no growth on Sabouraud dextrose agar. Without facilities for further fungal diagnostic work in Lao PDR, but with a probable clinical diagnosis of sporotrichosis, we sent one of the excised lesions to Taiwan for molecular analysis by previously described methods (*1**,**2*). Polymerase chain reaction (PCR) was negative for mycobacteria but positive for *Sporothrix schenckii*, the cause of sporotrichosis, and the diagnosis was confirmed by sequencing the 18S rRNA gene, which showed 100% identity to that of *S*. *schenckii* (*1**,**2*). The lesions resolved with 6 months of oral itraconazole therapy (100 mg every 12 h).

**Figure:**
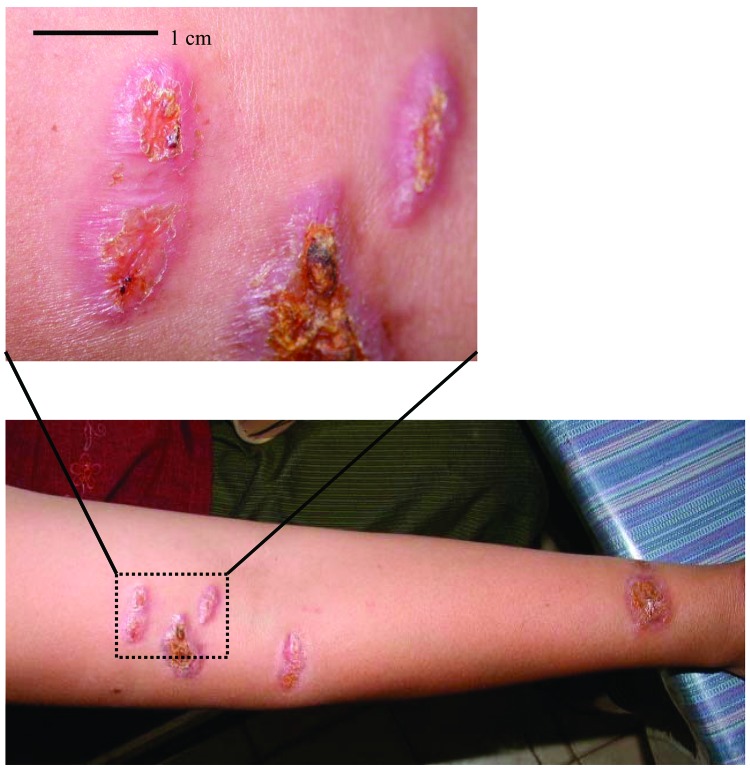
Lesions on the right arm of the patient.

*S*. *schenckii* is a dimorphic fungus found in soil, hay, decaying vegetation, and moss. Persons exposed to these environmental foci, such as farmers and gardeners, are especially at risk. Percutaneous inoculation is presumably the main method of infection, although inhalation and insect and mammal bites and scratches, especially from armadillos and cats, have been implicated (*3**,**4*). Our patient presumably contracted the fungus from the wood frame of the milling machine. In the 1940s, contamination from untreated wood was responsible for an epidemic that affected ≈3,000 gold miners in South Africa (from timbers in the mine). Lymphocutaneous sporotrichosis is the most frequent presentation, and the traditional treatments are oral saturated potassium iodide solution and local hyperthermia, but oral itraconazole for 3 to 6 months is now recommended (*3**,**4*).

Sporotrichosis has been described from North and South America, Europe, and Japan. In Asia and Australasia, it has been described from India (*5*), Taiwan (*1*), Australia (*6*), and Thailand (*7*), but apparently not from Laos, Cambodia, and Burma (Myanmar). Serologic evidence for human sporotrichosis infection is found in highland areas of southwest Vietnam (*8*). At least in part, the relative paucity of reports probably reflects the lack of sophisticated fungal diagnostic techniques in much of Southeast Asia. Some evidence suggests that sporotrichosis is more prevalent in tropical environments with relatively cool temperatures and high humidity such the Peruvian Andes (*9*), northwest India (*5*), southwestern Vietnam (*8*), and in Laos in the Plain of Jars. If this environmental association is correct, sporotrichosis may occur more extensively in the cooler humid areas of Asia, such as the highlands of China, Laos, Vietnam, and Burma. Sporotrichosis can disseminate in HIV-infected patients, and this syndrome may increase as the prevalence of HIV infection rises in these areas.

With 73% of the Lao population living on <US$2/day (*10*) and one accessible microbiologic culture laboratory in Laos, PCR is not an available local routine diagnostic technique. We were fortunate to have access to an overseas diagnostic facility, which allowed confirmation of the clinical diagnosis before the patient received a prolonged course of a drug with adverse effects and drug interactions.

Diagnosis by histopathologic examination and culture may be difficult, and identifying laboratories in different regions of the subtropics and tropics with an interest in diagnosis of sporotrichoid lesions and the capability to perform culture and PCR would facilitate the diagnosis and awareness of this disease. Itraconazole, which has become the drug of choice for lymphocutaneous sporotrichosis, is expensive. Saturated solution of potassium iodide is an inexpensive alternative and appears to be effective, although adverse effects occur frequently (*3**,**4*).
